# FEASIBILITY AND ACCURACY OF A POINT-OF-CARE COMMUNICATION FEEDBACK APP FOR NURSING HOME STAFF

**DOI:** 10.1093/geroni/igae098.3332

**Published:** 2024-12-31

**Authors:** Kristine Williams, Carissa Coleman, Jinxiang Hu, Xi Chen

**Affiliations:** University of Kansas Medical Center, Kansas City, Kansas, United States; University of Kansas Medical Center, Kansas City, Kansas, United States; University of Kansas Medical Center, Kansas City, Kansas, United States; University of Kansas Medical Center, Kansas City, Kansas, United States

## Abstract

Educating the long-term-care workforce in dementia care skills is critical. Research has established that reinforcing learned skills in practice is important for successful adoption. However, on-site, expert feedback is costly and not always feasible. The 3-hour Changing Talk (CHAT) educational intervention decreases staff elderspeak (communication that sounds like babytalk) which in turn, reduces negative behavioral responses of care recipients with dementia. We developed an automated feedback IOS application that records staff and uses natural language processing to analyze their speech, providing just-in-time feedback on their use of elderspeak diminutives (terms of endearment). This study evaluated feasibility and accuracy of the SPEEKO for Elderspeak app for point-of-care feedback on staff elderspeak communication. Long-term-care staff (n=17) self-recorded their communication during resident care. Amazon Translate language processing and a customized elderspeak algorithm were used to identify staff use of diminutives, a common part of elderspeak, in the recordings. Trained coders also completed manual transcription and psycholinguistic coding of diminutives in the recordings that were compared to the app results with chi-squared tests. The relationship of total diminutives comparing human and app identification was significant χ2=12.21, p<.001. Associations were significant for elderspeak diminutive word roots (“dear” χ2 =0.01, p=.004); “sweet” (χ2=20.20, p<.001); and “girl” (χ2=5.08, p=.02). Other terms were not significantly correlated (“honey” χ2=2.70, p=.10) and “baby” (χ2=0.85, p=.36). With improvements to overcome issues in accuracy of recording, transcription, and analyses, the SPEEKO for Elderspeak app has potential to provide self-monitoring feedback promoting implementation of communication skills in dementia care.

